# Bleeding phenotype and diagnostic characterization of patients with congenital platelet defects

**DOI:** 10.1002/ajh.25910

**Published:** 2020-07-14

**Authors:** Maaike W. Blaauwgeers, Marieke J.H.A. Kruip, Erik A.M. Beckers, Michiel Coppens, Jeroen Eikenboom, Karin P.M. van Galen, Rienk Y.J. Tamminga, Rolf T. Urbanus, Roger E.G. Schutgens

**Affiliations:** ^1^ Van Creveldkliniek, University Medical Center Utrecht University Utrecht Utrecht the Netherlands; ^2^ Department of Haematology Erasmus University Medical Center Rotterdam the Netherlands; ^3^ Department of Hematology Maastricht University Medical Center Maastricht the Netherlands; ^4^ Department of Vascular Medicine, Amsterdam Cardiovascular Sciences, Amsterdam University Medical Centers University of Amsterdam Amsterdam the Netherlands; ^5^ Department of Internal Medicine, division of Thrombosis and Haemostasis Leiden University Medical Center Leiden the Netherlands; ^6^ Department of Pediatric Hematology, Beatrix Children's Hospital University Medical Center Groningen Groningen the Netherlands; ^7^ Van Creveld Laboratory University Medical Center Utrecht, University Utrecht Utrecht the Netherlands

## Abstract

Phenotypic characterization of congenital platelet defects (CPDs) could help physicians recognize CPD subtypes and can inform on prognostic implications. We report the analyses of the bleeding phenotype and diagnostic characteristics of a large cohort of adult patients with a confirmed CPD. A total of 96 patients were analyzed and they were classified as Glanzmann thrombasthenia, Bernard‐Soulier syndrome, dense granule deficiency, defects in the ADP or thromboxane A2 (TxA2) pathway, isolated thrombocytopenia or complex abnormalities. The median ISTH‐BAT bleeding score was nine (IQR 5‐13). Heavy menstrual bleeding (HMB) (80%), post‐partum hemorrhage (74%), post‐operative bleeds (64%) and post‐dental extraction bleeds (57%) occurred most frequently. Rare bleeding symptoms were bleeds from the urinary tract (4%) and central nervous system (CNS) bleeds (2%). Domains with a large proportion of severe bleeds were CNS bleeding, HMB and post‐dental extraction bleeding. Glanzmann thrombasthenia and female sex were associated with a more severe bleeding phenotype.

## 1 INTRODUCTION

Congenital platelet disorders (CPDs) are rare bleeding disorders caused by congenital defects in platelet production or platelet function. Patients typically present with a mucocutaneous bleeding tendency. Common symptoms include epistaxis, unexplained or extensive bruising, oral cavity bleeds, heavy menstrual bleeding (HMB) and bleeding following a hemostatic challenge such as surgery, dental extraction and childbirth.^1^ So, CPDs are clinically heterogeneous; the frequency and severity of symptoms vary greatly among different types of CPDs, among patients with the same disorder and within patients over time.^2^

The bleeding phenotype can be evaluated with the ISTH Bleeding Assessment Tool (ISTH‐BAT). The ISTH‐BAT was designed to underscore the importance of repetitive minor bleeding in addition to more severe bleeds and can be used in all hemorrhagic disorders.^3^ The ISTH‐BAT is primarily designed as a screening tool. Since the ISTH‐BAT documents large variety of bleeding symptoms, it is also used for the phenotyping of patients.^4^, ^5^

Very few studies have reported the bleeding phenotype in adult patients with CPDs^6^ and most studies focused on a few specific types of CPD^7^, ^8^ or a specific mutation.^9^, ^10^ Phenotypic characterization of the whole spectrum of CPDs is necessary, since this could help physicians recognize CPD subtypes and inform new patients on prognostic implications regarding their bleeding phenotype. In this study, we aimed to evaluate the bleeding phenotype of a large cohort of adult patients with CPDs and to search for correlations between the bleeding score and different laboratory phenotypes of CPDs.

## 2 METHODS

### 2.1 Participant selection

Data were derived from patients included in the “Thrombocytopathy in the Netherlands” (TiN) study; a nationwide cross‐sectional study to collect data on clinical features, functional assays and genetics in a population of patients with or suspected for a CPD. Patients were included in the TiN study when von Willebrand disease or a coagulation factor deficiency were excluded and when they were previously diagnosed with a CPD, they had previously abnormal platelet count or function test results, or they exhibited a predominantly mucocutaneous bleeding tendency compatible with a platelet function disorder. Within the TiN study, a CPD was confirmed when abnormal platelet count or function was found on at least two occasions, of which one was in our diagnostic laboratory. For the current evaluation, we included only TiN patients in whom a CPD diagnosis was confirmed.

### 2.2 Bleeding phenotype

The ISTH‐BAT was used for evaluation of the patients' bleeding symptoms and was administered by an experienced physician prior to platelet function testing. It contains questions on 14 domains: epistaxis, cutaneous bleeding, bleeding from minor wounds, urinary tract bleeding, gastrointestinal bleeding, oral cavity bleeding, post‐dental extraction bleeding, post‐operative bleeding, heavy menstrual bleeding (HMB), post‐partum hemorrhage (PPH), muscle hematomas, hemarthrosis, central nervous system (CNS) bleeding and one final domain on other bleeding symptoms. Each domain was scored on a scale ranging from zero to four points. We classified a bleeding symptom as severe when the domain score was three or higher, since this indicates that the bleeding symptom required medical treatment. The total of all domains resulted in a bleeding score ranging from 0‐56. The cut‐off values for an abnormal bleeding score are >3 for men and > 5 for women.^11^

### 2.3 Laboratory assessment

Laboratory tests were performed for platelet count, aggregation in response to four agonists (ADP, arachidonic acid, collagen, ristocetin), platelet ADP and ATP content, surface receptor expression with flow cytometry and whole‐exome sequencing (WES) with a selected 76‐gene panel (Table S1). Platelet morphology, gray platelets and leukocyte inclusion bodies were assessed in a peripheral blood smear. The cut‐off value for abnormal platelet aggregation was determined for every agonist and was based on the 2.5th percentile, plus the coefficient of variation of 52 healthy donors (Table S2). The cut‐off value for abnormal platelet receptor expression was determined based on the 2.5th percentile of 49 healthy donors (Table S3). Dense granule deficiency was diagnosed when the ADP content was lower than 1.4 μmol/10^11^ platelets, based on the 2.5th percentile of 49 healthy donors. In line with the American College of Medical Genetics guidelines, a genetic variant was stated to be causal when a (likely) pathogenic variant (class four or five, respectively)^12^ was identified in one or more of the selected genes that corresponded to the platelet phenotype.

### 2.4 Statistical analysis

Statistical analyses were performed with IBM SPSS Statistics 25, GraphPad Prism software version 6 and RStudio version 0.99. Descriptive results for continuous variables were presented as medians (IQR), and categorical variables were presented as frequencies (percentages). The difference in bleeding score between types of CPDs, and between men and women was evaluated with linear regression analysis. Correlations between bleeding score and number of abnormal agonists in light transmission aggregometry (LTA), and between bleeding score and ADP content were calculated with non‐parametric Spearman's rank correlation. Correlation coefficients (ρ) of 0.20‐0.39 were considered weak, 0.40‐0.59 moderate, 0.60‐0.79 strong and >0.8 very strong.^13^ Only moderate or stronger correlations were considered relevant.

## 3 RESULTS

A CPD diagnosis was confirmed in 96 TiN patients. The majority of patients were women (61/96, 64%) (Table 1). The median age was 38 years (IQR 28‐53) for women and 40 years (IQR 26‐57) for men. The median bleeding score was 9 (IQR 7‐15) for women and 6 (IQR 3‐12) for men.

**TABLE 1:** Patient characteristics

	N = 96
Sex	
Women, n (%)	61 (64)
Age	
Women, median (IQR)	38 (28–53)
Men, median (IQR)	40 (28‐57)
Bleeding score	
Women, median (IQR)	9 (7–15)
Men, median (IQR)	6 (3–12)
Type of CPD	
ADP pathway defect, n (%)	22 (23)
Bernard‐Soulier syndrome, n (%)	4 (4)
Complex abnormality^a^, n (%)	6 (6)
Dense granule deficiency, n (%)	13 (14)
Glanzmann thrombasthenia, n (%)	14 (14)
Isolated thrombocytopenia, n (%)	22 (23)
TxA2 pathway defect, n (%)	15 (16)

Abbreviations: CPD, congenital platelet defect; IQR, interquartile range; TxA2, thromboxane A2.

^a^ Complex abnormality was diagnosed when the patterns of platelet function defects did not support the diagnosis of a particular type of CPD.

### 3.1 Classification of patients based on diagnostic characteristics

#### 3.1.1 Glanzmann thrombasthenia

**FIGURE 1:**
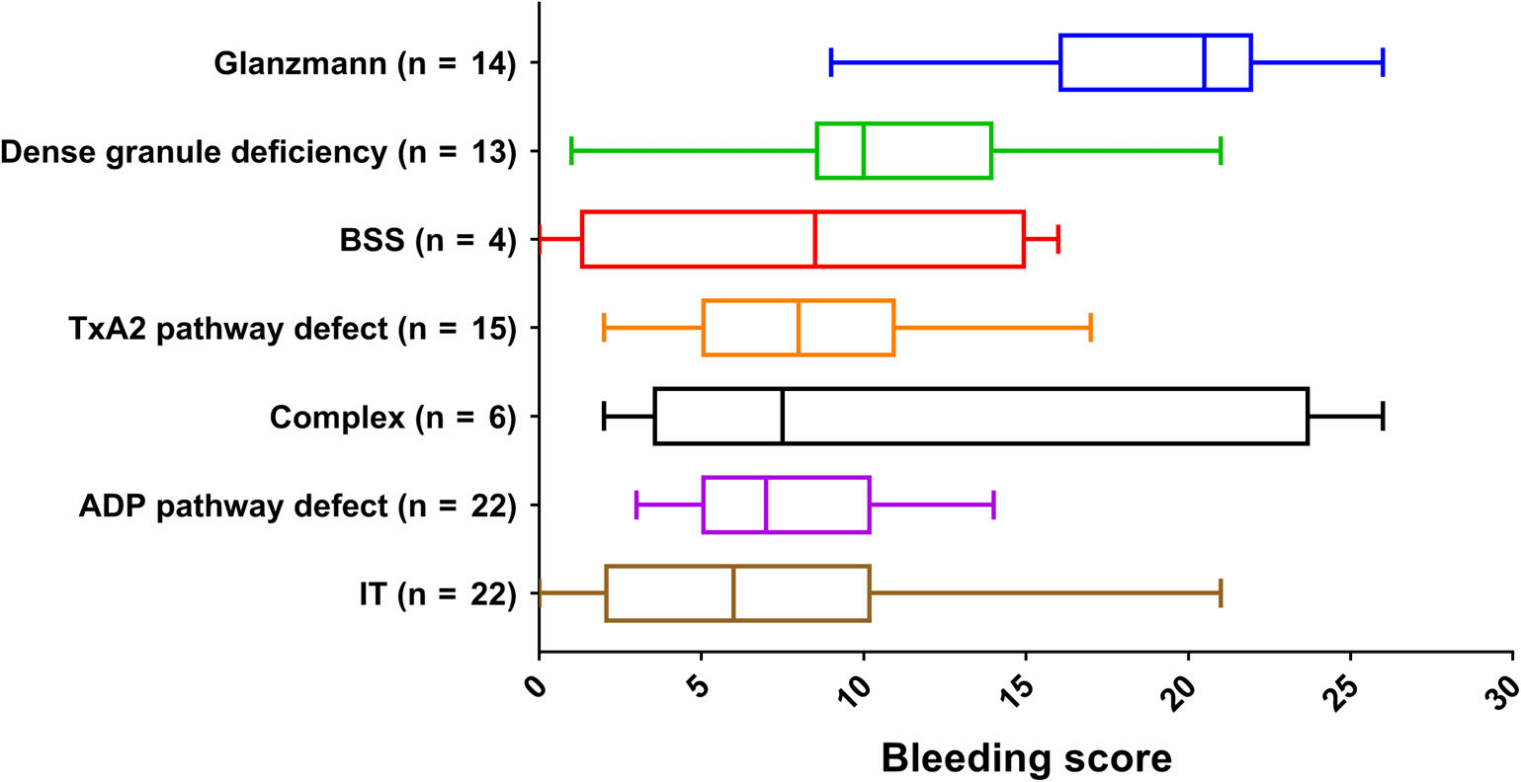
Bleeding score per type of congenital platelet defect. Boxes represent median and interquartile range, whiskers represent minimum and maximum. BSS, Bernard‐Soulier syndrome; IT, isolated thrombocytopenia; TxA2, thromboxane A2 [Color figure can be viewed at wileyonlinelibrary.com]

Glanzmann thrombasthenia (GT) was diagnosed in 14 patients based on decreased or absent aggregation in response to ADP, arachidonic acid and collagen and decreased αIIbβ3 expression. The median bleeding score was 21 (IQR 16‐22) (Figure 1). The median platelet count was 183 × 10^9^/L (IQR 133‐226) and in 5/14 patients the platelet count was below the normal range. The median αIIbβ3 expression was 1.9% (IQR 1.4% ‐ 3.3%). Eleven patients had type 1 GT, one patient had type 2 GT and two patients had variant type GT. Genetic mutations were identified in all 14 patients.

#### 3.1.2 Bernard‐Soulier syndrome

Bernard‐Soulier syndrome (BSS) was diagnosed in four patients based on a decreased aggregation in response to ristocetin and decreased GP1b‐V‐IX expression. The median bleeding score was nine (IQR 1‐15). All patients had macrothrombocytopenia with a median platelet count of 53 × 10^9^/L (IQR 16‐80) and median MPV of 16.3 fL (IQR 15.9 fL‐16.4 fL). The median GP1b‐V‐IX expression was 24.2% (IQR 16.5% ‐ 26.9%) and genetic analysis confirmed BSS in all patients.

#### 3.1.3 Dense granule deficiency

Dense granule deficiency was diagnosed in 13 patients based on platelet ADP content below 1.4 μmol/10^11^ platelets. The median bleeding score was 10 (IQR 9‐14). The median platelet count was 165 × 10^9^/L (IQR 69‐303) and in 6/13 patients the platelet count was below the normal range. The median platelet ADP content was 0.9 μmol/10^11^ platelets (IQR 0.5‐1.2). The LTA results were abnormal in 6/13 patients, but did not reveal a specific pattern. Genetic mutations were identified in five patients.

#### 3.1.4 ADP pathway defect

An isolated ADP pathway defect was diagnosed in 22 patients based on decreased aggregation in response to ADP. A normal aggregation in response to arachidonic acid differentiates them from patients with a thromboxane A2 (TxA2) pathway defect (Figure S1A). The median bleeding score was seven (IQR 5‐10). The median aggregation in response to ADP was 54% (IQR 47% ‐ 61%). Platelet ADP content was normal in all patients. Genetic mutations were identified in three patients.

#### 3.1.5 Thromboxane A2 pathway defect

A TxA2 pathway defect was diagnosed in 15 patients based on decreased aggregation in response to arachidonic acid (AA), with or without defective aggregation in response to other agonists (Figure S1B). The median bleeding score was eight (IQR 5‐11). The median aggregation in response to AA was 10% (IQR 4% ‐ 14 %). Platelet ADP content was normal in all patients. Genetic mutations were identified in two patients.

#### 3.1.6 Isolated thrombocytopenia

An isolated thrombocytopenia (IT) was diagnosed in 22 patients based on a low platelet count, normal platelet function and normal platelet ADP content. An acquired thrombocytopenia was ruled out based on negative glycoprotein specific antibodies and thorough anamnesis, including familiy history and previous treatment for thrombocytopenia, with or without response. The median bleeding score was six (IQR 2‐10). The median platelet count was 80 × 10^9^/L (IQR 49‐126) and median MPV 9.3 fL (IQR 8.5fL‐12.2 fL). Genetic mutations were identified in two patients.

#### 3.1.7 Complex abnormalities

A complex abnormality was diagnosed in six patients. A complex abnormality was diagnosed when the patterns of platelet function defects in LTA did not support the diagnosis of a particular type of CPD as described above. The median bleeding score was eight (IQR 4‐24). No genetic mutations were identified in this subgroup.

### 3.2 Bleeding symptoms in patients with CPDs

**FIGURE 2:**
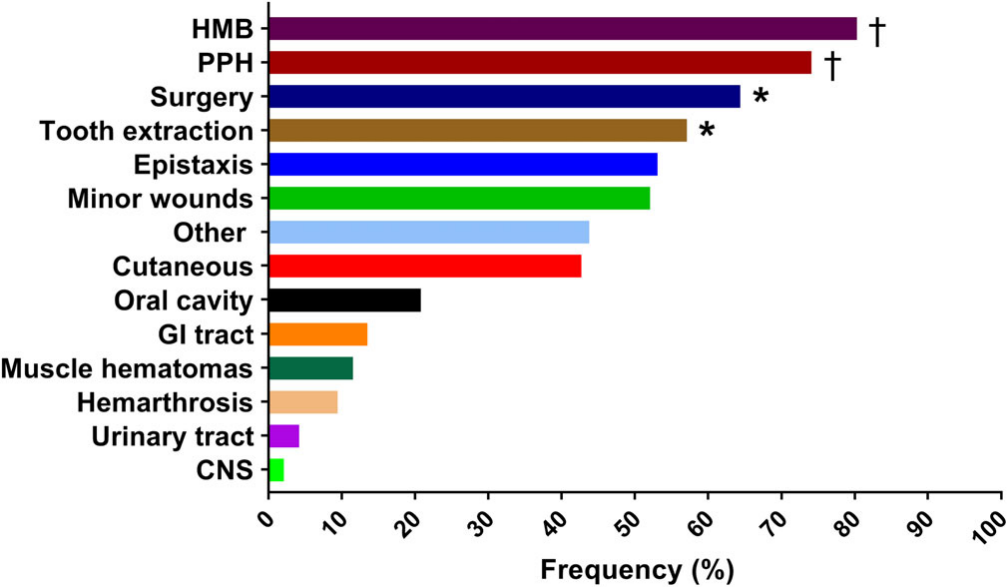
Prevalence of bleeding symptoms in patients with congenital platelet defects (CPDs). Proportion of CPD patients who experienced the bleeding symptom (ISTH‐BAT domain score ≥ 1). *Frequencies are based on patients who underwent tooth extraction (n = 63) or surgery (n = 73). †Frequencies are based on women who have been menstruating (n = 61) or gave birth (n = 27). CNS, central nervous system; GI, gastrointestinal; HMB, heavy menstrual bleeding; PPH, postpartum hemorrhage [Color figure can be viewed at wileyonlinelibrary.com]

Most frequently occurring bleeding symptoms were HMB (49/61, 81% of women), PPH (20/27, 74% of women who gave birth) and post‐operative bleeds (47/73, 64% of patients who underwent surgery) (Figure 2). Rare bleeding symptoms were bleeds from the urinary tract (4/96, 4%) and CNS bleeds (2/96, 2%).

**FIGURE 3:**
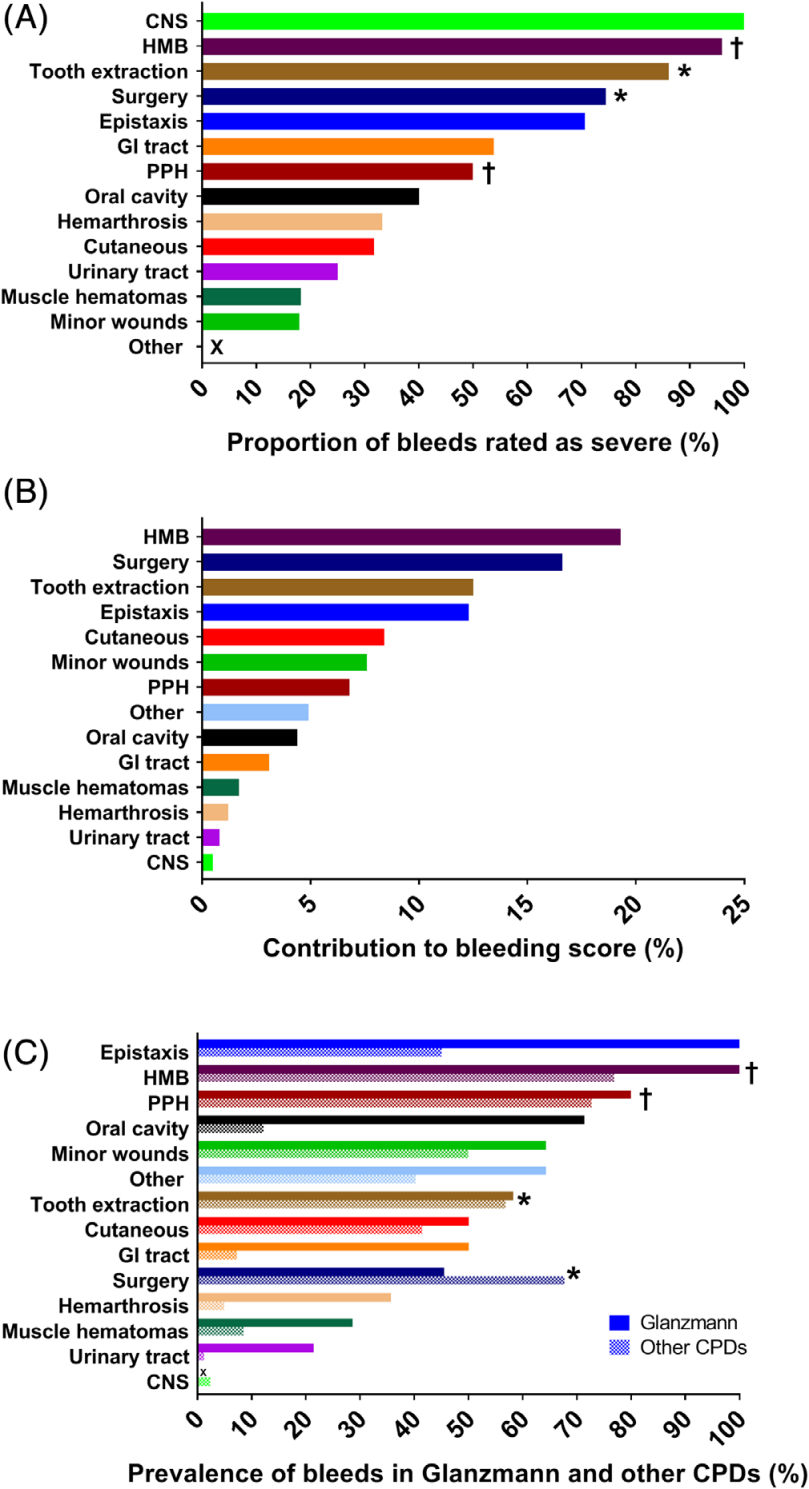
Prevalence of (severe) bleeding symptoms and contribution of bleeding symptoms to the bleeding score. A, Prevalence of severe bleeding in patients with CPDs. Proportion of CPD patients who experienced the bleeding symptom and in whom the bleeding symptom was severe (ISTH‐BAT domain score ≥ 3). B, Contribution of the bleeding symptoms to the bleeding score. The frequency and severity of the bleeding symptoms are combined to calculate the contribution of each domain to the sum of all bleeding scores. C, Prevalence of bleeding symptoms in patients with Glanzmann and patients with other types of CPDs. Proportion of Glanzmann patients (solid bars) and patients with other types of CPDs (striped bars) who experienced the bleeding symptom (ISTH‐BAT domain score ≥ 1). CNS, central nervous system; CPD, congenital platelet defect; GI, gastrointestinal; HMB, heavy menstrual bleeding; PPH, postpartum hemorrhage. *Frequencies are based on patients who underwent tooth extraction or surgery. †Frequencies are based on women who have been menstruating or gave birth [Color figure can be viewed at wileyonlinelibrary.com]

We classified a bleeding symptom as severe when the domain score was three or higher. Both CNS bleeds were classified as severe (Figure 3A). Other domains of the ISTH‐BAT with a large proportion of severe bleeds were HMB (47/49, 96% of women who have experienced HMB), post‐dental extraction bleeding (31/36, 86% of patients who experienced bleeding after tooth extraction) and post‐operative bleeding (35/47, 75% of patients who experienced bleeding after surgery). Bleeds from minor wounds were relatively common (50/96, 52%), as were bleeds in the “other” category (42/96, 44%), but these bleeds were rarely severe: nine of 50 patients (18%) had severe bleeds from minor wounds, none of 42 patients reported severe “other” bleeds.

By combining the frequency and severity of the bleeding symptoms, the contribution of each domain to the sum of all bleeding scores can be calculated (Figure 3B). Bleeding symptoms with the largest contribution to the bleeding score were HMB (19%), post‐operative bleeding (17%), post‐dental extraction bleeding (13%) and epistaxis (12%).

The bleeding score was significantly higher in women than in men (β 3.5, 95% CI 0.9 to 6.1). When the bleeding score was calculated without the domains HMB and PPH, there was no significant difference (β 0.1, 95% CI −2.1 to 2.4).

### 3.3 Bleeding symptoms according to type of CPD

Adjusted for age and sex, patients with GT had a significantly higher bleeding score than patients with other types of CPDs. As compared to other types of CPDs, GT patients more frequently (*P* < .01) experienced epistaxis (100% vs 45%), urinary tract bleeds (21% vs 1%), gastrointestinal bleeds (50% vs 7%), oral cavity bleeds (71% vs 12%) and hemarthrosis (36% vs 5%) (Figure 3C). For the other types of CPDs, the bleeding phenotype was not distinctive. The bleeding phenotypes of CPDs other than GT were similar.

### 3.4 Association between bleeding score and laboratory measurements

Correlations between the bleeding score and ADP, arachidonic acid and collagen aggregation were weak to moderate (ρ −0.31, −0.40 and − 0.50 respectively). The bleeding score showed a moderate correlation with the number of abnormal agonists in LTA (ρ 0.45, 95% CI 0.23 to 0.63). When GT patients were not taken into account, the bleeding score did not correlate with the number of abnormal agonists in LTA (ρ 0.16, 95% CI −0.10 to 0.42). Also, the bleeding score did not correlate with a lower platelet ADP content (ρ 0.03, 95% CI −0.19 to 0.25), nor with a lower platelet count (ρ 0.17, 95% CI −0.03 to 0.36).

## 4 DISCUSSION

This is the first study to report the bleeding phenotype and diagnostic characteristics of a large cohort of adult patients with CPDs. Patients were classified as Glanzmann thrombasthenia, Bernard‐Soulier syndrome, dense granule deficiency, defects in the ADP or thromboxane A2 (TxA2) pathway, isolated thrombocytopenia or complex abnormalities. Most frequently occurring bleeding symptoms were HMB, PPH and post‐operative bleeds. Glanzmann thrombasthenia and female sex were associated with a more severe bleeding phenotype. The bleeding score was not correlated with the number of abnormal agonists in LTA, nor with a lower platelet ADP content or lower platelet count.

Eighty percent of women reported HMB and in 96% of cases it was classified as severe. Moreover, HMB accounted for almost 20% of the sum of all bleeding scores. And, when the bleeding score in women was calculated without the domains HMB and postpartum hemorrhage, there was no difference between men and women. Taken together, this indicates that HMB is a considerable health problem in women with CPDs. Serious bleeding complications like CNS bleeds, gastrointestinal bleeds and hemarthrosis were rare, although more common in patients with GT as compared to other types of CPDs.

Very few studies described the bleeding phenotype of patients with CPDs. One previous study in a large cohort of patients with CPDs showed that epistaxis and cutaneous bleeds most frequently occurred, and that epistaxis and oral cavity bleeding were the most severe bleeding symptoms.^6^ Glanzmann thrombasthenia was widely represented in their study population (40%) and their study population had fewer women included (57%) as compared to ours (64%). Another previous study reported bleeding scores in a cohort of patients with CPDs,^14^ but they did not report on the frequency of bleeding symptoms. Their study population consisted of mostly children and the Pediatric Bleeding Questionnaire was used. One study reported the bleeding phenotype of patients with Von Willebrand disease,^4^ a primary hemostasis defect with similar symptoms. They used the Tosetto bleeding score^15^ to assess bleeding symptoms and their patients most frequently reported HMB, cutaneous bleeds and prolonged bleeding from minor wounds.

It is debatable whether the ISTH‐BAT is the most suitable tool for assessing the severity of bleeding symptoms, since domains of the ISTH‐BAT saturate easily. Patients who have experienced a single bleed after surgery that required DDAVP treatment score the same as patients with multiple bleeds after surgery that required several transfusions. Also, CNS bleeds are always classified as severe, since the score is either zero, three or four for that domain. Despite its limitations, the ISTH‐BAT is at the moment the best tool to assess bleeding symptoms, because it takes both the frequency and the severity of the whole spectrum of bleeding symptoms into account.

In our study, there was a risk for selection bias. Patients with a clinical suspicion for a CPD were referred for inclusion in the TiN study and it is likely that the referral rate was higher for patients with clinically important bleeds, such as HMB or bleeding after a hemostatic challenge, than for patients with clinically less important bleeds, such as cutaneous bleeds. This might explain the high prevalence of these bleeding symptoms in our population. In addition, for some patients it was not reported whether they had undergone tooth extraction. When patients reported no bleeding after tooth extraction, in 6% of cases it was not reported whether they had undergone tooth extraction at all. These patients were not included in the analysis for that domain, which could have led to a slight overestimation of the prevalence. Also, the ISTH‐BAT was not completed at the time of diagnosis for some patients, but at the time of inclusion in the study. These patients possibly had more hemostatic challenges, since they had more time to develop bleeding symptoms, resulting in a higher bleeding score. Some of these bleeds might have required treatment, which would also have led to an increase in the bleeding score.

The strength of our study is the inclusion of a large number of CPD patients. To the best of our knowledge, this is to first study to evaluate the bleeding phenotype and diagnostic characteristics in a large cohort of adult patients with various subtypes of CPDs. Insight into the bleeding phenotype of these patients can lead to better counseling of patients regarding the prognostic implications of their bleeding phenotype. It will create more awareness on the clinical picture of CPDs among doctors and in society. We incorporated explicit diagnostic criteria for CPDs based on expert opinion. Standardized diagnostic criteria will reduce under‐diagnosis and misdiagnosis in patients. Moreover, it will allow cluster analysis in patients with similar phenotypes and further unraveling of the pathophysiology of platelet defects.

In conclusion, in CPD patients most frequently occuring symptoms are HMB and bleeding after a hemostatic challenge. Glanzmann thrombasthenia and female sex are associated with a more severe bleeding phenotype.

## Supporting information


**Table S1** Genes included in the WES gene panel for molecular screening of primary hemostatic disorders
**Table S2.** Cut‐off values for light transmission aggregometry (LTA).
**Table S3.** Cut‐off values for platelet receptor expression with flow cytometry.
**Figure S1**. Aggregation in response to ADP, arachidonic acid (AA), collagen and ristocetin in patients with (A) an ADP pathway defect and (B) a TxA2 pathway defect. Bars represent reference values (range) for healthy controls.
