# Supplementary material for: Bleeding phenotype and diagnostic characterization of patients with congenital platelet defects
Source: Am J Hematol. 2020 Jul 14;95(10):1142–7. doi: 10.1002/ajh.25910 (PMC7540397; doi:10.1002/ajh.25910)
Supplement: Supplementary file 1 — Table S1 Genes included in the WES gene panel for molecular screening of primary hemostatic disorders Table S2. Cut‐off values for light transmission aggregometry (LTA). Table S3. Cut‐off values for platelet receptor expression with flow cytometry. Figure S1. Aggregation in response to ADP, arachidonic acid (AA), collagen and ristocetin in patients with (A) an ADP pathway defect and (B) a TxA2 pathway defect. Bars represent reference values (range) for healthy controls. [file AJH-95-1142-s001.docx]

**SUPPLEMENTARY TABLES**

**Table S1.** Genes included in the WES gene panel for molecular screening of primary hemostatic disorders

| **Target protein** | **Gene** | **Description** | **Gene** | **Description** |
| --- | --- | --- | --- | --- |
| Platelet agonist receptors | *ADRA2A* | G-protein coupled receptors | *GP9* | Bernard Soulier syndrome |
|  | *ADRA2B* | G-protein coupled receptors | *ITGA2* | Bleeding disorder, platelet type 9 |
|  | *CD36* | Bleeding disorder, platelet type 10 | *ITGA2B* | Glanzmann thrombasthenia |
|  | *F2R* | G-protein coupled receptors | *ITGB1* | Bleeding disorder, platelet type 9 |
|  | *F2R13* | G-protein coupled receptors | *ITGB3* | Glanzmann thrombasthenia |
|  | *GP1BA* | Bernard Soulier syndrome | *P2RY12* | Bleeding disorder, platelet type 8 |
|  | *GP1BB* | Bernard Soulier syndrome | *TBXA2R* | Bleeding disorder, platelet type 13 |
|  | *GP6* | Bleeding disorder, platelet type 11 |  |  |
| Platelet granules | *AP3B1* | Hermansky-Pudlak syndrome 2 | *LYST* | Chediak-Higashi syndrome |
|  | *BLOC1S3* | Hermansky-Pudlak syndrome 8 | *MLPH* | Griscelli syndrome |
|  | *BLOC1S6* | Hermansky-Pudlak syndrome 9 | *MYO5A* | Griscelli syndrome |
|  | *DTNBP1* | Hermansky-Pudlak syndrome 7 | *NBEAL2* | Gray platelet syndrome |
|  | *HPS1* | Hermansky-Pudlak syndrome 1 | *PLAU* | Quebec platelet disorder |
|  | *HPS3* | Hermansky-Pudlak syndrome 3 | *RAB27A* | Griscelli syndrome |
|  | *HPS4* | Hermansky-Pudlak syndrome 4 | *VPS33B* | ARC syndrome |
|  | *HPS5* | Hermansky-Pudlak syndrome 5 | *VIPAS39A* | ARC syndrome |
|  | *HPS6* | Hermansky-Pudlak syndrome 6 |  |  |
| Signal transduction | *PLA2G4A* | Phospholipase A2 deficiency | *RGS2* | G-protein signaling |
|  | *PTGS1* | Bleeding disorder, platelet type 12 | *TBXAS1* | Bleeding disorder, platelet type 14 |
|  | *RASGRP2* | Bleeding disorder, platelet type 18 |  |  |
| Transcription factors | *CYCS* | Thrombocytopenia 4 | *HOXA11* | CTRUS syndrome |
|  | *ETV6* | Thrombocytopenia 5 | *MECOM* | CTRUS syndrome |
|  | *FLI1* | Bleeding disorder, platelet type 21 | *RBM8A* | TAR syndrome |
|  | *GATA1* | GATA1-related disorder | *RUNX1* | FPD/AML |
|  | *GFI1B* | Bleeding disorder, platelet type 17 | *STIM1* | Stormorken syndrome |
| Cytoskeletal and structural proteins | *ABCG5* | Sitosterolemia | *FYB* | CARST syndrome |
|  | *ABCG8* | Sitosterolemia | *MASTL* | Thrombocytopenia 2 |
|  | *ACTN1* | Bleeding disorder, platelet type 15 | *MYH9* | MYH9-related disorders |
|  | *ANKRD26* | Thrombocytopenia 2 | *PRKACG* | Bleeding disorder, platelet type 19 |
|  | *CDC42* | Takenouchi-Kosaki syndrome | *TUBB1* | TUBB1-related macrothrombocytopenia |
|  | *FERMT3* | Leukocyte adhesion deficiency III | *WAS* | Wiskott-Aldrich syndrome |
|  | *FLNA* | Filaminopathy |  |  |
| Procoagulant disorders | *ANO6* | Scott syndrome |  |  |
| Collagen disorders | *COL1A1* | Ehlers-Danlos syndrome | *COL5A1* | Ehlers-Danlos syndrome |
|  | *COL3A1* | Ehlers-Danlos syndrome | *COL5A2* | Ehlers-Danlos syndrome |
| Blood vessel abnormalities | *ACVRL1* | Hereditary telangiectasia | *ENG* | Hereditary telangiectasia |
| Fibrinogen disorders | *FGA* | Dys/hypo/afibrinogenemia | *FGG* | Dys/hypo/afibrinogenemia |
|  | *FGB* | Dys/hypo/afibrinogenemia |  |  |
| Other | *GBA* | Gaucher disease | *SLFN14* | Bleeding disorder, platelet type 20 |
|  | *GNE* | GNE myopathy | *THPO* | Thrombocytemia 1 |
|  | *MPL* | CAMT syndrome | *VWF* | von Willebrand disease |

ARC, arthrogryposis, renal dysfunction and cholestasis; CAMT, congenital amegakaryocytic thrombocytopenia; CARST, congenital autosomal recessive small-platelet thrombocytopenia; CTRUS, congenital thrombocytopenia with radioulnar synostosis; FPD/AML, familial platelet disorder with propensity to acute myelogenous leukemia; TAR, thrombocytopenia and absent radius; WES, whole exome sequencing.

**Table S2.** Cut-off values for light transmission aggregometry (LTA).

| **LTA** | **ADP 2.5** | **ADP 5.0** | **Arachidonic acid** | **Collagen 1.0** | **Collagen 4.0** | **Ristocetin** |
| --- | --- | --- | --- | --- | --- | --- |
| 2.5^th^ percentile + CV | 36 | 73 | 60 | 67 | 78 | 75 |

CV, coefficient of variance

**Table S3.** Cut-off values for platelet receptor expression with flow cytometry.

| **Receptor expression** | α2bβ3 | α2β1 | GP1b-V-IX | GP6 |
| --- | --- | --- | --- | --- |
| 2.5^th^ percentile | 32 | 57 | 60 | 33 |

**SUPPLEMENTARY FIGURES**

**Figure S1**. Aggregation in response to ADP, arachidonic acid (AA), collagen and ristocetin in patients with (A) an ADP pathway defect and (B) a TxA2 pathway defect. Bars represent reference values (range) for healthy controls.
